# Haploinsufficiency for *BRCA1 *is associated with normal levels of DNA nucleotide excision repair in breast tissue and blood lymphocytes

**DOI:** 10.1186/1471-2350-6-26

**Published:** 2005-06-14

**Authors:** Jean J Latimer, Wendy S Rubinstein, Jennifer M Johnson, Amal Kanbour-Shakir, Victor G Vogel, Stephen G Grant

**Affiliations:** 1 Department of Obstetrics, Gynecology and Reproductive Sciences, School of Medicine, University of Pittsburgh, Pittsburgh, PA, USA; 2 Biochemistry and Molecular Genetics Program, School of Medicine, University of Pittsburgh, Pittsburgh, PA, USA; 3 Research Institute, Magee-Womens Hospital, Pittsburgh, PA, USA; 4 Department of Medicine, Northwestern University Feinberg School of Medicine, Chicago, IL, USA; 5 Evanston Northwestern Healthcare Center for Medical Genetics, Evanston, IL, USA; 6 Department of Pathology, School of Medicine, University of Pittsburgh, Pittsburgh, PA, USA; 7 Department of Medicine, School of Medicine, University of Pittsburgh, Pittsburgh, PA, USA; 8 Department of Environmental and Occupational Health, Graduate School of Public Health, University of Pittsburgh, Pittsburgh, PA, USA

## Abstract

**Background:**

Screening mammography has had a positive impact on breast cancer mortality but cannot detect all breast tumors. In a small study, we confirmed that low power magnetic resonance imaging (MRI) could identify mammographically undetectable tumors by applying it to a high risk population. Tumors detected by this new technology could have unique etiologies and/or presentations, and may represent an increasing proportion of clinical practice as new screening methods are validated and applied. A very important aspect of this etiology is genomic instability, which is associated with the loss of activity of the breast cancer-predisposing genes* BRCA1* and *BRCA2*.  In sporadic breast cancer, however, there is evidence for the involvement of a different pathway of DNA repair, nucleotide excision repair (NER), which remediates lesions that cause a distortion of the DNA helix, including DNA cross-links.

**Case presentation:**

We describe a breast cancer patient with a mammographically undetectable stage I tumor identified in our MRI screening study. She was originally considered to be at high risk due to the familial occurrence of breast and other types of cancer, and after diagnosis was confirmed as a carrier of a Q1200X mutation in the *BRCA1 *gene. In vitro analysis of her normal breast tissue showed no differences in growth rate or differentiation potential from disease-free controls. Analysis of cultured blood lymphocyte and breast epithelial cell samples with the unscheduled DNA synthesis (UDS) assay revealed no deficiency in NER.

**Conclusion:**

As new breast cancer screening methods become available and cost effective, patients such as this one will constitute an increasing proportion of the incident population, so it is important to determine whether they differ from current patients in any clinically important ways. Despite her status as a *BRCA1* mutation carrier, and her mammographically dense breast tissue, we did not find increased cell proliferation or deficient differentiation potential in breast epithelial cells from this patient which might have contributed to her cancer susceptibility. Although NER deficiency has been demonstrated repeatedly in blood samples from sporadic breast cancer patients, analysis of blood cultured lymphocytes and breast epithelial cells for this patient proves definitively that heterozygosity for inactivation of *BRCA1 *does not intrinsically confer this type of genetic instability. These data suggest that the mechanism of genomic instability driving the carcinogenic process may be fundamentally different in hereditary and sporadic breast cancer, resulting in different genotoxic susceptibilities, oncogene mutations, and a different molecular pathogenesis.

## Background

A reduction in breast cancer mortality has been observed in recent years that has been partially attributed to the widespread adoption of screening mammography [1]. Traditional screening mammography, however, fails to detect 15% of incident cancers [2]. New, complementary imaging techniques are therefore under development that may increase the accuracy of primary screening. We performed a small study to validate the use of low power magnetic resonance imaging (MRI) to prospectively detect breast alterations and malignancy and to determine the feasibility of applying this technique to a high-risk population [3]. We present here a subject from that study whose early stage tumor was not detectable by mammography.

This patient was enrolled in the screening study due to her family history of breast and other neoplasias. After tumor diagnosis, she was determined to be heterozygous for a putative inactivating mutation in the *BRCA1 *gene. In addition, she had dense breast tissue, an impediment to mammography that is in itself a risk factor for breast cancer [4]. Breast development and lactational differentiation also appear to individually modify breast cancer risk, with early term pregnancy conferring a persistent protective effect [5]. Exposure to ionizing radiation, while a lifetime risk factor for breast cancer, appears to be more dangerous when it occurs during alveolar differentiation of the breast at adolescence [6]. Using a novel tissue engineering system [7], we therefore examined the growth and differentiation of normal breast epithelial samples from this patient via live-cell imaging.

The *BRCA1 *hereditary breast cancer gene has been shown to be involved in DNA double strand break repair [8,9]. DNA repair defects have also been identified in the peripheral blood cells of sporadic breast cancer patients [10-13], but, in this case, it seems to involve a different pathway of DNA repair, nucleotide excision repair (NER) [14-16]. We have extended this observation of NER deficiency to the tumor itself, as well as the adjoining non-diseased normal breast tissue [17]. NER is a complex pathway of DNA repair [18] normally associated with removal of pyrimidine-pyrimidine intrastrand crosslinks (“dimers”) caused by exposure to UV light.  NER deficiency is the basis of hereditary xeroderma pigmentosum (XP) [19], a disease with a 1200-fold increase in incidence of skin cancer [20].  The signal for activation of the NER pathway is actually very general; any lesion causing a distortion in the DNA helix, including crosslinks caused by oxidative radicals, certain types of mismatches (purine-purine or pyrimidine-pyrimidine) and so called “bulky” adducts caused by phase I metabolism of polyaromatic hydrocarbons [21]. It has recently been shown that *BRCA1 *expression can enhance NER activity, although this analysis was not performed in breast cells [22,23]. We therefore applied the functional unscheduled DNA synthesis (UDS) assay for NER capacity to multiple samples of normal tissue from this patient, to determine whether haploinsufficiency for *BRCA1 *was a mechanism of NER deficiency. We have developed a method to reliably culture non-diseased breast tissue (with a success rate of 100%) and breast tumors (with a success rate of 85%) [7,17].

## Case presentation

We describe a breast cancer patient whose tumor was detected by MRI. She was enrolled into a pilot screening study of low power MRI due to her familial risk. She had mammographically dense breasts and her tumor was undetectable mammographically.

### Patient description

**Figure 1:**
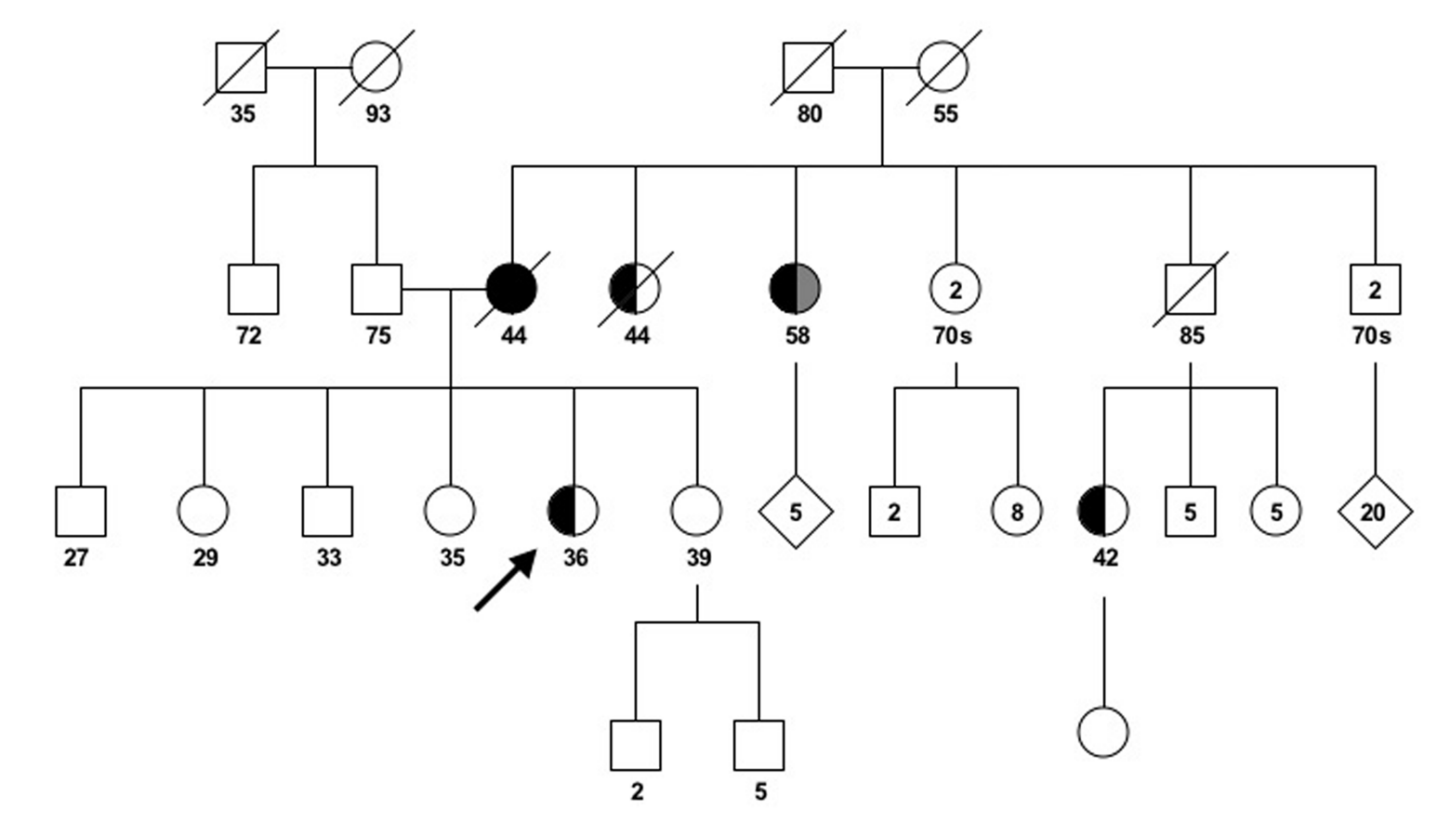
**Pedigree of the patient (indicated by arrow). **She, one maternal aunt and one maternal cousin had breast cancer diagnosed at 36, 44 and 41 years old, respectively, as indicated by the half-filled symbols, and her aunt died of the disease. Her cousin underwent lumpectomy followed by chemotherapy, radiotherapy and is presently on tamoxifen. Her mother had breast cancer in both breasts, diagnosed at ages 41 and 42, as indicated by the completely filled symbol. She underwent bilateral mastectomy and hysterectomy followed by chemotherapy and radiotherapy and died of the disease at age 44. A second maternal aunt was diagnosed with colon cancer at age 52 (light half-filled symbol) and breast cancer at age 55 (dark half-filled symbol). Based on this pattern of familial cancer the patient was considered to be at high risk of developing breast cancer and was entered into the low power MRI screening validation and feasibility study. Following her diagnosis, she was confirmed as carrying a Q1200X mutation in the *BRCA1 *gene.

The patient was a 35.7 year old woman who presented with a very strong family history of breast cancer as depicted in Figure 1, and negative physical and mammographic examination. She had extremely dense breast tissue bilaterally by mammography as well as fibrocystic breast tissue by physical examination. She had no previous personal history of breast biopsy or abnormal mammograms.

### Risk profile

The 5 year breast cancer risk for this patient as calculated by the BRCAPRO model was 5.7%, and her probability of being a *BRCA1 *or *BRCA2 *carrier was 0.47. The Gail model risk assessment was calculated using the following information: Race-Caucasian; Age-35; Age at first menses-12; Age at first live birth-nulliparous; Number of first-degree relatives with breast cancer-2; Number of previous breast biopsies-0. The calculated 5 year Gail risk was 1.0% and her lifetime risk was 31.3%.

### Genetic testing

Following genetic counseling, the patient elected to undergo DNA sequencing of the *BRCA1 *and *BRCA2 *genes, which revealed a Q1200X truncation mutation in one of her *BRCA1 *alleles. The C to T mutation at codon 1200 in exon 11 results in the change of the amino acid glutamine to a stop codon with resulting protein truncation and loss of function. Exon 11 is the largest exon in *BRCA1 *and has the highest frequency of reported mutations. The Q1200X mutation has been independently observed several times [24].

### Imaging

The bilateral screening mammogram was compared to previous films from another hospital. The breast tissue was described as heterogeneously dense, thus lowering the sensitivity. There were no masses, significant calcifications or other findings and the mammogram was interpreted as negative bilaterally. A one-year follow-up was recommended.

The patient was then MRI scanned as previously described [3], with pre- and post-gadolinium enhancement images evaluating both breasts simultaneously in the axial plane. In the upper-outer left breast there was a small (approximately 1 cm), round, well-demarcated enhancing lesion. This lesion was seen on both the initial delay after contrast injection and the delayed contrast enhanced subtraction images. The lesion appeared to accumulate contrast to a greater extent on the delayed subtraction images with an additional lesion adjacent to the first. In the medial aspect of the mid right breast, there were several small punctate areas of enhancement on both the immediate and delayed subtraction views. Also in the right breast just above the nipple level medial and close to the chest wall an additional enhancing lesion was seen. This lesion was approximately 1.5 cm, round, well-demarcated and continued to accumulate contrast on the delayed subtraction images. This lesion appeared to have a small non-enhancing septation.

### Core biopsies

**Figure 2:**
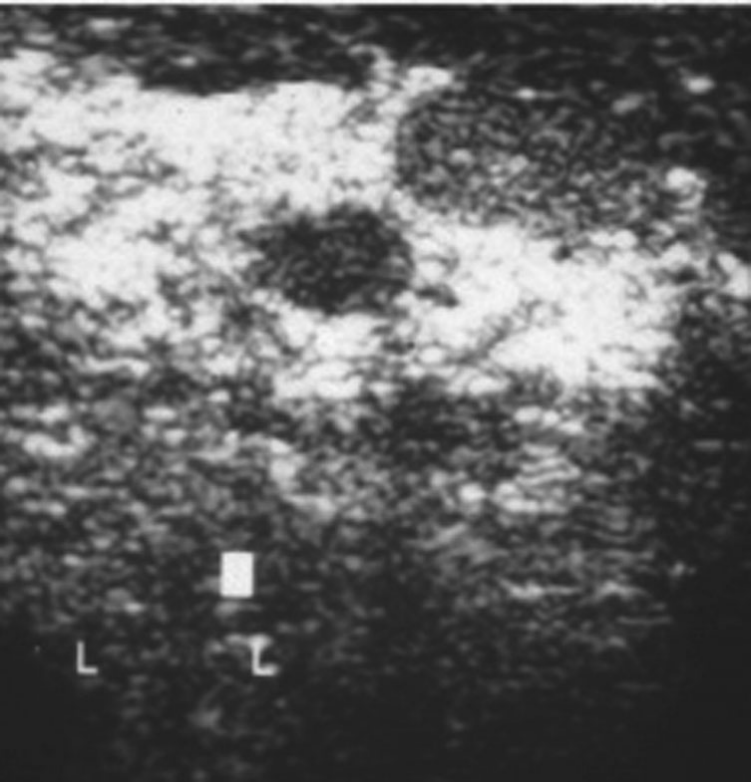
**Ultrasound of the MRI-detected lesion. **Following MRI, the patient was scheduled for ultrasound to identify the questionable lesions seen on MRI for possible core biopsy. Under ultrasound the lesion of concern was identified and biopsied at the 1:00 location in the left breast. Additionally, one lesion seen by MRI in the right breast at the 4:00 location was identified and biopsied.

Under ultrasound, the lesion of concern in the left breast was identified and biopsied, as well as one lesion in the right breast (Figure 2). The core biopsy of the left breast revealed infiltrating ductal carcinoma in 2 of 5 core fragments; high nuclear grade, with no lymphatic invasion seen. The core biopsy of the right breast demonstrated benign pathology, specifically, fibrosis with focal ductal epithelial hyperplasia.

### Final pathology, treatment plan and outcome

Although a surgical candidate for lumpectomy and radiation, the patient chose to undergo left modified radical mastectomy with left axillary lymph node dissection and contralateral prophylactic total mastectomy because of her genetic risk status. The pathology in the left breast was consistent with the imaging and core biopsy in size and description. Tumor size was 8 mm in greatest dimension, nuclear grade III, ER/PR and Her2/neu negative, and the nodal status (0/4) was negative (stage TIaN0M0). The patient underwent 4 cycles of chemotherapy and has been reportedly healthy since. Because of the positive *BRCA1 *mutation results, she subsequently underwent prophylactic bilateral salpingo-oophorectomy.

### Live-cell analysis of tissue explant cultures

**Figure 3:**
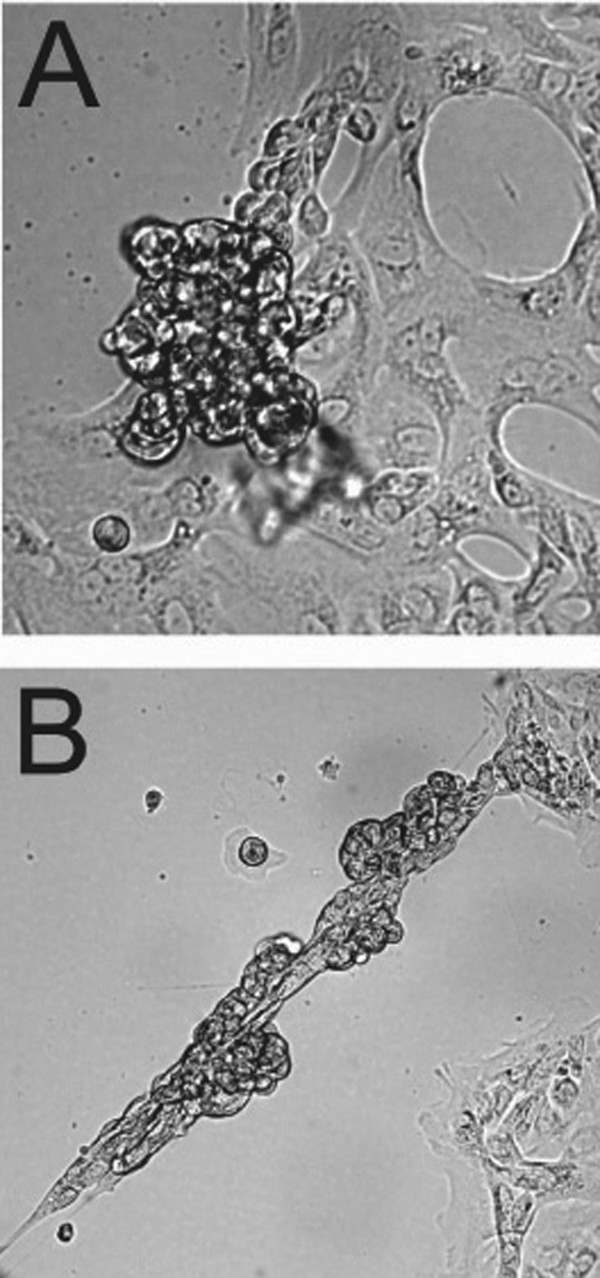
**Micrographs of the non-diseased primary human mammary epithelial cultures (HMEC) from the *BRCA1 *mutation carrier. **A) Contralateral breast – A cluster of epithelial cells called a mammosphere is shown on the left center of the image sitting on a field of fibroblasts. B) Ipsilateral breast – The original fresh tissue block from which this culture was derived was located 4 cm from the infiltrating ductal carcinoma. The structure shown is a cluster of rounded epithelial cells manifesting a column configuration called "pre-ductal linearization". Both images were captured under Differential Interference Contrast (DIC) optics on a Zeiss Axiovert 100 microscope at a total of 140x magnification.

A number of life history factors have been associated with breast cancer incidence that are widely interpreted as representing lifetime exposure of the breast tissue to estrogen-induced mitogenesis [25]. An alternative interpretation, based on epithelial cell differentiation, suggests that lactational differentiation, such as occurs during term pregnancy, confers resistance to carcinogenesis [26,27]. We have developed a novel human mammary epithelial (HME) tissue engineering system wherein many aspects of organotypic differentiation are reiterated in vitro [28]. In this system, breast epithelial cells initially retain cell-to-cell contact while they proliferate, then undergo an architectural reorganization, first to form three-dimensional mammospheres, and later vast networks of branching ductal and lobular structures. Tumor and some pre-neoplastic samples fail to form such architecture. Normal tissue from this patient, who is both a *BRCA1 *mutation carrier and has dense breasts, was evaluated to determine whether either of these factors affected de novo differentiation in this system. Four discrete pieces of fresh tissue were provided for live-cell analysis from each of the patient's ipsilateral and contralateral breasts. In the case of the ipsilateral breast, this tissue was provided at increasing distance from the tumor margin in 1 cm increments. All of these normal samples attached and grew in our culture system and were examined for cell-to-cell interactions and morphology over a period of one month. In the context of breast reduction explant cultures from 22 patients with no breast disease, these patient samples manifested typical mixtures of fibroblastic and epithelial cells. After several days in culture without passaging, the epithelial cells began to self-organize, initially forming three-dimensional mammospheres (Figure 3A), and, after 2 weeks in culture, more complex pre-ductal linear columns of epithelial cells (Figure 3B). The tissue explants from both breasts showed similar patterns of behavior (Figure 3). Tissue cultured from a contemporaneous disease-free control and the contralateral breast of a sporadic breast cancer patient showed similar morphology and architecture (data not shown).

### Cell growth kinetics

It has been suggested that the association between breast density and risk of breast cancer is due to increased cell proliferation [29]. One measure of cell growth and viability is the S-phase index (SPI) or the percentage of cells incorporating radiolabeled thymidine over a specific incubation period (in our case, 2 hours). In a previous study with 22 normal breast reduction epithelium [BRE] cultures we observed a wide range of proliferation rates, with SPI ranging from a low of 0.2% to a high of 46.0% (mean of 18.3 ± 2.6%) [30]. The contemporaneous control sample from a disease-free breast reduction patient had an SPI of 30.9%, at the higher end of this normal range. The ipsilateral and contralateral tissue samples from the hereditary breast cancer patient exhibited SPI of 26.6% and 26.2%, respectively, placing them at slightly over the 70^th ^percentile for growth rate. The contralateral sample from the sporadic breast cancer patient had an SPI of 17.0%, placing it slightly under the 50^th ^percentile. Thus, all of these breast cancer patient samples appeared to grow well in our system, with SPI well within the range of our normal samples. The similarity of the SPI values from the two samples from the *BRCA1 *mutation carrier does not appear to be accidental; the chances of selecting two samples from the normal population with values as close or closer is very small (*P *= 0.026).

### Functional analysis of NER capacity

Peripheral blood lymphocytes and normal breast epithelial tissue from the hereditary cancer patient were then cultured for performance of the functional UDS assay, which requires living cells for radiolabel incorporation during DNA repair synthesis following UV exposure. This assay is diagnostic for the inherited cancer-prone disease XP, where it is usually performed in lymphocytes or skin fibroblasts. Our novel HME tissue engineering system allows us to apply the assay to breast epithelial cells, and we have previously demonstrated tissue-specificity in the NER capacity of these cells in normal samples from patients undergoing breast reduction mammoplasty [30]. Patient data is therefore expressed relative to the average of our breast reduction controls.

**Figure 4:**
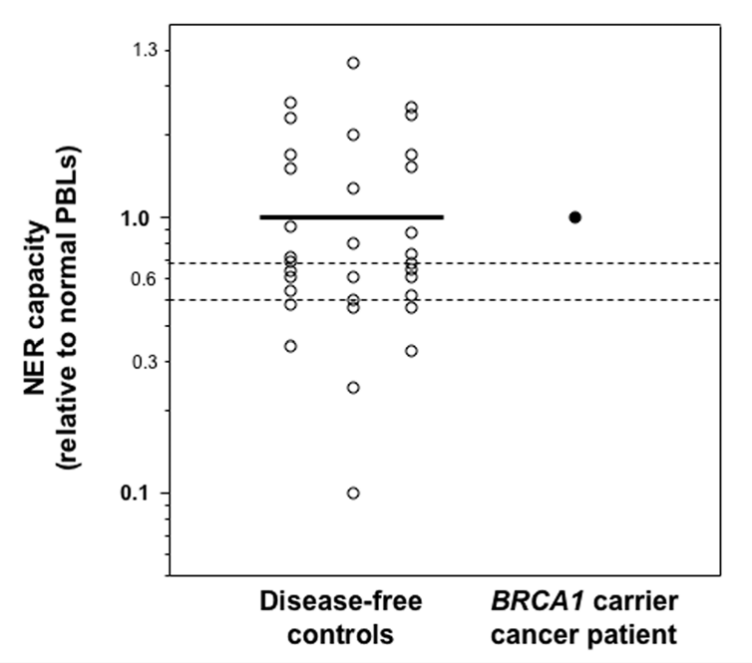
**Comparison of the NER capacity of a PBL sample from our *BRCA1 *mutation carrier patient with those of a population of disease-free controls. **The dark horizontal line indicates the average for the normal population, while the dotted lines indicate upper limits for residual NER activity in patients with the hereditary NER deficiency disease XP (0.50) and the cut-off established in our breast tissue study that identified tumors with high sensitivity and specificity (0.70).

Analysis of cultured blood lymphocytes from the patient established that they had normal NER capacity (99.6% of the average of our 33 normal samples) (Figure 4). This is well above the cut-off established in our sporadic breast cancer population, < 70% average normal activity, which when applied to our cases and controls yielded a significant odds ratio of 37.4 [31]. A trend towards age dependence had been noted in the analysis of the UDS data of the normal controls (*P *= 0.059) [30]; addition of the patient sample supports this trend, but it still fails to reach significance (*P *= 0.056).

**Figure 5:**
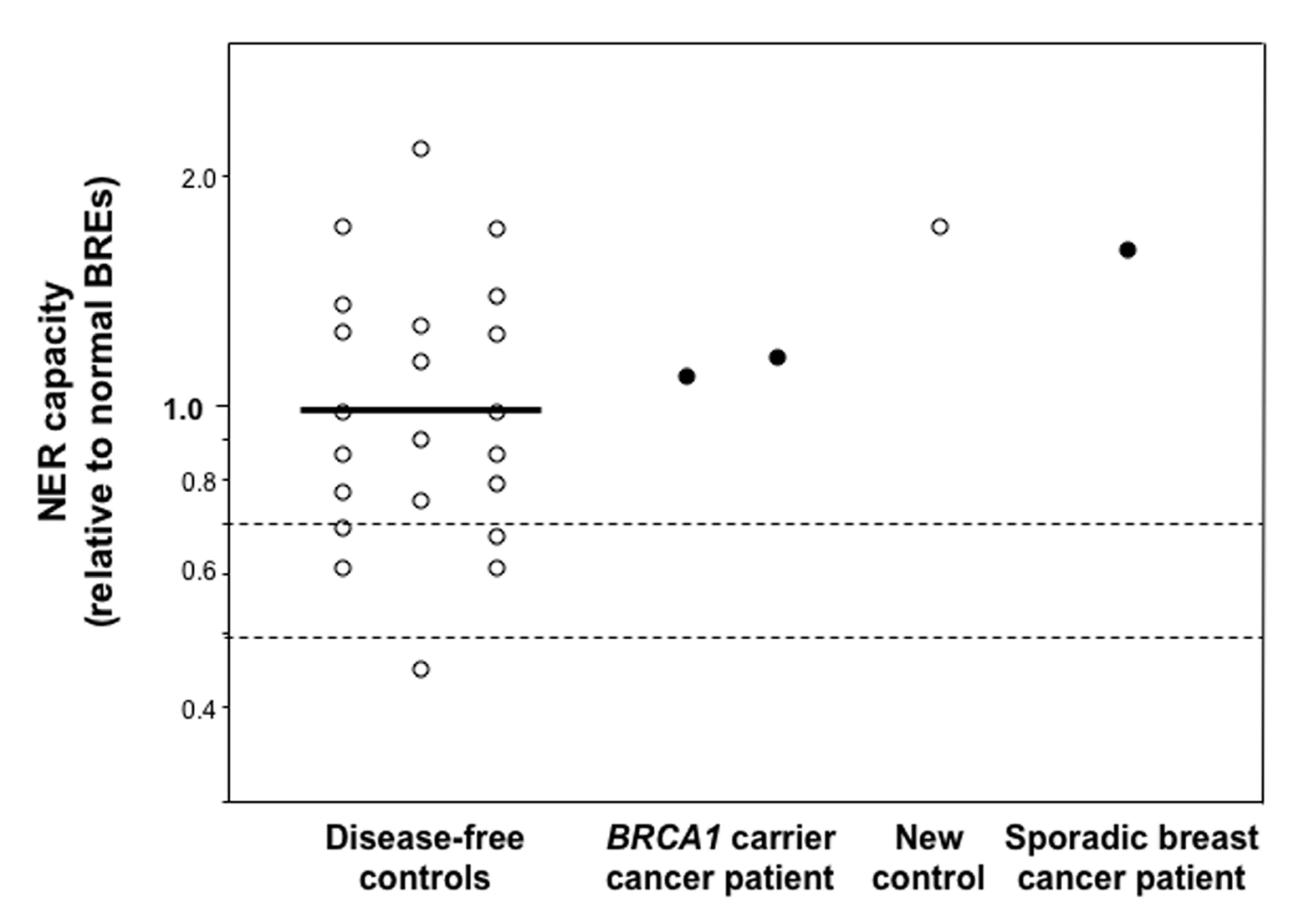
**Comparison of the NER capacities of two samples of normal breast epithelium from our *BRCA1 *mutation carrier patient with those of a population of disease-free controls who underwent breast reduction mammoplasty. **The dark horizontal line indicates the average for the normal population of breast reduction epithelium (BRE), while the dotted lines indicate upper limits for residual NER activity in patients with the hereditary NER deficiency disease XP (0.50) and the cut-off established in our breast tissue study that identified tumors with high sensitivity and specificity (0.70). The patient sample on the left was derived from the ipsilateral (left) breast, while the sample on the right was from the contralateral (right) breast.

The functional NER assay was then applied to the contemporaneous disease-free breast reduction control sample, one sample each from the ipsilateral and contralateral breasts of the patient, and to a sample from the contralateral breast of an apparently sporadic breast cancer patient. The NER of the BRE non-diseased control was 1.82 times the average of our normal data set for this tissue and within the range of normal. The NER capacity of the ipsilateral breast epithelial sample was 1.05 times the average of our population of BRE controls, clearly exhibiting no overt DNA repair deficiency (Figure 5). The contralateral sample was very similar, with an NER capacity of 1.17 times BRE normal. Although the NER values of these two samples from the same patient are similar, they are not close enough to distinguish themselves as coming from the same individual (*P *= 0.16). The NER capacity of the contralateral sample from the sporadic breast cancer patient was 1.62 times the average of the BRE controls, also in the normal range.

Our earlier analysis of NER in our normal population revealed no effects of age or cell proliferation (as represented by the SPI). All of these additional patient samples are consistent with those results.

## Discussion

At least two types of breast tumors are not accurately detected by traditional screening mammography: "interval" tumors that arise quickly between screenings, and tumors whose density is not sufficient to distinguish them from the surrounding normal tissue. The latter situation is more likely to occur in women with dense normal breast tissue, which, in turn, is more typical of younger women. Thus, mammographically undetectable tumors may have a number of characteristics, such as fast growth, low density, early onset and/or occurrence in dense breasts that might distinguish them from mammographically detectable tumors in terms of molecular etiology and clinical parameters of prognosis and response. The present patient had an early onset breast tumor, but had both hereditary susceptibility due to her *BRCA1 *mutation and dense breasts, so her presentation is not unusual in this context. It is possible that breast tumors detected by complementary screening methods in the future will demonstrate unique clinical and molecular features, when it becomes feasible to perform such screening in the general population.

Since the *BRCA1 *gene product is known to play a role in DNA double strand break repair [8,9], it has been suggested that decreased repair capacity is the basis of the breast cancer predisposition observed in mutation carriers [32-35]. Such a cellular phenotype has been difficult to demonstrate, however [36-39]. An alternate possibility is that the mutation affects the growth or differentiation of breast epithelial cells in a manner consistent with cancer susceptibility. It has been suggested that dense breast tissue is indicative of generalized hyperproliferation that might promote oncogenesis [29]. Our findings show that all 8 samples, derived from both the involved and the uninvolved breasts of a hereditary breast cancer patient develop normal epithelial architecture in vitro, implying that the epithelial/stromal (paracrine) interactions necessary for the development of this complex architecture are intact and normal in *BRCA1 *heterozygotes despite their greater risk of breast cancer. The SPI results also indicate that this non-diseased epithelial tissue falls into the typical range of normal for BRE control cultures and is demonstrating typical growth in our HME tissue engineering system.

NER deficiency is most often associated with XP, sensitivity to UV-induced DNA damage and skin cancer [18-21]. The NER deficiency of XP patients is manifested in other tissues, however, as shown by their high spontaneous frequency of mutation in blood lymphocytes [40] and the occurrence of other types of tumors [41]. The observation that sporadic breast cancer patients have low levels of NER in peripheral lymphocytes suggests that sporadic breast cancer is associated with constitutively low levels of NER [14-16]. Our results from a single patient demonstrate, however, that while overexpression of *BRCA1 *may enhance NER [22], haploinsufficiency for this gene does not necessarily result in detectable NER deficiency. Since it is clear that genomic instability is a necessary prerequisite for the completion of the complex multi-step carcinogenic pathway(s) involved in breast cancer, a fundamental difference in the mechanisms of genomic instability arising in hereditary and sporadic breast tumors would be likely to translate into fundamentally different patterns of molecular pathogenesis that could impact on clinical management.

The relative NER capacities of tumor and normal tissue may have important practical implications.  If breast tumors from hereditary patients exhibit NER deficiency similar to that observed in sporadic patients, while their normal tissues exhibit normal levels of this type of DNA repair, then the tumors would be hypersensitive to a range of chemotherapeutic drugs, including alkylating agents (cyclosphosphamide), cross-linking agents (cis-platinum) and bulky DNA adducting agents (melphalan).  Individualization of chemotherapy based on some aspect of NER expression is being pursued in colon [42], testicular [43,44] and ovarian cancer [45].

## Conclusion

This patient and her tumor represent the vanguard of a new population of early stage breast cancer patients that will be increasingly diagnosed as new screening technologies complementary to mammography are validated and become practicable. We have shown that low power MRI can detect a stage I tumor in dense breast tissue; the same technology can also impact upon interval tumors by staggering the procedure with mammography rather than applying them coincidently. Although we did not observe obvious differences in the growth rate or differentiation potential of the dense breast tissue from this patient, we cannot rule out the possibility that some or all of the tumors detectable only by complementary screening procedures will differ from the present clinical experience in important ways. Our live-cell analysis takes a step toward defining cellular characteristics that may be useful for cancer risk assessment, but we are only beginning to investigate the possibilities of the system. It may be that different growth conditions, or induction with genotoxic or estrogenic agents, will allow for the greater differentiation of breast tissue and tumor behaviours. This technique also allows for the application of functional assays to patient samples, as exemplified in this report by the UDS assay for NER capacity. Those UDS results, although from a single patient, demonstrate definitively that the constitutively low NER capacities reported in several sporadic breast populations do not arise as a pleiomorphic effect of *BRCA1 *haploinsufficency. Thus, the basis of genetic instability, a fundamental element in breast carcinogenesis, may differ between sporadic and hereditary breast tumors. This results in different susceptibilities to inducing agents, mutations in different sets of oncogenes and tumor suppressor genes, and, ultimately, tumors of different molecular etiology that express different clinically relevant phenotypes.

## Methods

### Patients and controls

The patient was a 35.7 year old woman with strong family history of breast cancer recruited into a clinical trial of MRI screening for young woman at high risk for breast cancer with dense breast tissue [3]. Gadolinium enhancement images revealed a small 1 cm lesion in the upper-outer quadrant of the left breast, identified pathologically as an infiltrating ductal carcinoma. The patient underwent a modified radical mastectomy of the left breast and chose to also undergo a contralateral prophylactic total mastectomy. Blood and tissue were obtained for analysis with consent under Magee-Womens Hospital (of the University of Pittsburgh Medical Center) IRB # MWH-94-108.

Data from this hereditary breast cancer patient were compared to that from two additional patients as well as previously published controls. The first new control patient was a 20 year old women undergoing breast reduction mammoplasty. The second contemporaneous control patient was a 36 year old woman undergoing cosmetic surgery on her contralateral breast two years after successful lumpectomy to remove an apparently sporadic stage IIA breast tumor (2.5 cm, negative for estrogen and progesterone receptors, 13 lymph nodes negative). She had undergone standard radiotherapy and chemotherapy with adriamycin and cyclophosphamide. Histopathological analysis confirmed that the breast tissue from both of these control patients was free of cancer and within the acceptable histological range of normal.

### Patient tissue culture and analysis

Fresh tissues from the patient were obtained within 5 hours of surgery. After pathological evaluation, excess tissue not needed for diagnosis was placed into DMEM containing 10% fetal calf serum and 3x antibiotic antimycotic (Sigma, St. Louis, MO) at 4°C. This tissue was then processed as described in Latimer *et al*. [30] and placed into culture on a diluted form of matrigel (1:1 with DMEM) in the novel MWRIα medium [7].

Eight samples of the principal patient's tissue were obtained for culture after bilateral mastectomy surgery. We were not able to obtain a sample of her tumor, because it was utilized entirely for clinical diagnosis. We were able to obtain 4 pieces of histologically normal non-tumor adjacent tissue at increasing 1 cm intervals from the tumor margin from her left (ipsilateral) breast. In addition, we obtained 4 similar pieces of fresh tissue from her contralateral breast. All were placed into primary explant (HME) culture.

For analysis of cell growth and in vitro differentiation, explants were cultured and imaged every second day using a digital Hamamatsu Orca camera for 30–60 days. Images were analyzed on a Macintosh G4 computer using QED imaging software (Media Cybernetics, Inc., Silver Spring, MD).

### Control tissue cultures

Breast reduction mammoplasty tissues were obtained from patients ages 20–70 at Magee-Womens Hospital under the above IRB. A neighboring piece of mammoplasty tissue (from the same 0.25 cm^2 ^sample) to that placed into primary culture was fixed and processed in paraffin. These sections were examined by a pathologist to verify the histological features and normality of the tissue. Breast tissue was processed as previously described [30]. Tissue was rinsed three times in PBS containing antibiotics, disaggregated and placed into MWRIα medium [7] on a thin coat of matrigel. Peripheral blood lymphocytes (PBLs) were obtained with consent from normal healthy control subjects ages 20–50 working at Magee-Womens Hospital or students at the University of Pittsburgh. Foreskin fibroblast (FF) tissue was obtained as discarded tissue from newborn infants after circumcision and utilized between passages 7 and 10. These control populations have been previously described in greater detail [30,46]. Breast tissue samples from the two new control patients were processed in the same manner.

### Analysis of S-phase indices

Primary cultures of mammary tissue, established 10–14 days, were labeled with ^3^H-thymidine for a period of 2 hours followed by a chase with cold thymidine for 2 hours and then processed for autoradiography. After a 10–12 day exposure, slides were processed and analyzed by two independent, blinded scorers who evaluated the tissue samples for the percentage of cells in S phase (characterized by complete coverage of the nucleus with silver grains).

### Unscheduled DNA synthesis

NER was measured by autoradiography of unscheduled DNA synthesis after UV damage (UDS) [47,48]. After a total of 10–14 days in culture, without passaging, cultures were irradiated with UV light at 254 nm at a mean fluence of 1.2 Joules/m^2 ^for 12 seconds in the absence of culture medium, for a total dose of 14 J/m^2^. Each sample was represented by at least two chamber slides. One chamber of each 2-chamber slide was shielded from the UV dose to be used as an unirradiated control sample. Primary cultures had not reached confluence and were still actively growing at the time the UDS assay was performed. Control FF were plated subconfluently 2 days before the UDS assay to insure that they also were not in a quiescent state brought on by confluence. After UV exposure, all cultures were incubated in medium supplemented with 10 μCi ml [^3^H]methyl-thymidine (~80 Ci mmol^-1^) (PerkinElmer Life Sciences, Boston, MA) for 2 hours at 37°C. Labeling medium was then replaced with unlabeled chasing medium containing 10^-3 ^M non-radioactive thymidine (Sigma) and incubated for a further 2 hours to clear radioactive label from the intracellular nucleotide pools. After incubation in the post-labeling medium, cells were fixed in 1X SSC, 33% acetic acid in ethanol, followed by 70% ethanol and finally rinsed in 4% perchloric acid over night at 4°C. All slides were dried and subsequently dipped in photographic emulsion (Kodak type NTB2) and exposed for 10 to 14 days in complete darkness at 4°C.

The length of exposure of emulsion was determined in each experiment by preparing FF "tester" slides. After 10–12 days these tester slides were developed and grain counting was performed. If the nuclei over the foreskin fibroblasts averaged 50 or more grains per nucleus, then the rest of the experimental slides were developed. If the grain count was below this level, the remaining slides were left to expose 1–3 days longer before being developed.

### Grain counting

After photographic development of emulsion, all slides were stained with Giemsa, then examined at a total magnification of 1000X on a Zeiss Axioskop under oil emersion for grains located immediately over the nuclei of non-S phase cells [48]. Local background grain counts were evaluated in each microscopic field over an area the same size as a representative nucleus, and this total was subtracted from the grain count of each nucleus in that field. The average number of grains per nucleus was quantified for each side of the chamber slide, both unirradiated and irradiated. The final NER value for each slide was calculated by subtracting the unirradiated mean (grains per nucleus) from the irradiated mean (grains per nucleus), after the initial subtraction of local background in each field. NER was initially expressed as a percentage of the activity of concurrently analyzed FF. Four FF slides were scored per experiment, by an average of three counters. 200 nuclei were counted per slide, for a total of 800, with an average of 61.6 grains/nucleus. Six slides were evaluated for the patient's PBL sample, two by each of three counters. An average of 195 nuclei were scored per slide (for a total of almost 1200), with an average of 7.5 grains/nucleus. Four slides were counted for the contemporaneous breast reduction control, two each by two counters. There were an average of 200 nuclei per slide and 14.1 grains/nucleus. Six slides were scored from the patient's ipsilateral breast tissue sample, two by each of three independent counters, and five slides were counted from the contralateral sample, again by three independent counters. An average of just over 100 nuclei were evaluated per slide for each sample, for a total of almost 600 nuclei for the ipsilateral sample and over 500 for the contralateral sample. As the NER capacities indicate, these samples had very similar counts; about 35 grains/nucleus for the ipsilateral sample and 28 grains/nucleus for the contralateral sample. Finally, four slides were counted from the contralateral sample of a sporadic breast cancer patient, by three counters. There were an average of 200 nuclei per slide and 29.4 grains per nucleus.

### Statistical analysis

To ensure accuracy and guard against transcription errors, raw grain counts from the UDS assay were processed independently in duplicate, once using StatView (version 5.0.1, SAS Institute, Inc., Cary, NC), and once using the Data Analysis Toolpack of the Excel 2001 spreadsheet program (Microsoft Corp., Redmond, WA). The final count from slides of the same cell type within the same experiment and developed the same day were averaged together and expressed as a percentage of concurrently analyzed FF. These results were then normalized by comparison to the average for the tissue type control population [48].

## Competing interests

The authors declare that they have no competing interests.

## Authors' contributions

JJL conceived of the study, executed it, and drafted the manuscript. WSR recruited and consented the patient, provided clinical samples and information. JMJ evaluated the UDS assay and analyzed the data. AKS performed the histopathological analysis of the tissue. VGV participated in the study design and data interpretation. SGG participated in the design and coordination of the study and helped to draft the manuscript.

## Pre-publication history

The pre-publication history for this paper can be accessed here:
